# Differential sedimentary evolution of typical aulacogens of Meso-Neoproterozoic in North China craton

**DOI:** 10.1038/s41598-024-54027-7

**Published:** 2024-02-22

**Authors:** Cong Tan, Qiqi Lyu, Tongshan Wang, Qiufen Li, Hua Jiang, Xue Yan

**Affiliations:** 1 PetroChina Research Institute of Petroleum Exploration & Development, Beijing, 100083 China; 2 School of Geosciences, Yangtze University, Wuhan, 430100 China

**Keywords:** Sedimentary evolution, Meso-Neoproterozoic, Yanliao aulacogen, Xiong'er aulacogen, North China craton, Environmental sciences, Solid Earth sciences

## Abstract

Many countries and regions in the world have obtained industrial oil flow in the Meso-Neoproterozoic sedimentary strata and formed commercial exploitation in recent years. The development horizon of high-quality source rocks in the Proterozoic in North China can be compared with the international, indicating that the Meso-Neoproterozoic in North China has great exploration potential. The sedimentary characteristics of typical aulacogen in multiple cratons in the Meso-Neoproterozoic North China Craton are compared and studied by using field outcrop data, drilling data and analysis and test data, aiming to provide sedimentary support for the prediction of oil and gas distribution and evaluation of exploration field in the Meso-Neoproterozoic in this area. The results show that there are four sedimentary systems in the study area, including Marine clastic rock sedimentary system, Marine carbonate sedimentary system, Marine-continental transitional facies sedimentary system and glacial sedimentary system. They are divided into seven sedimentary facies types: barrier coastal facies, non-barrier coast facies, shallow shelf facies, carbonate platform facies, reef facies, fan delta facies and glacial facies, and further divided into 15 subfacies and 21 microfacies. On this basis, the Meso-Neoproterozoic sedimentary filling sequences of two typical aulacogens, Yanliao and Xiong 'er, in the study area are clarified, showing that the formation time of each sedimentary filling sequence stage of different aulacogens is different, and the rock characteristics, lithology combination, lithologic structure, contact relationship, vertical sequence and sedimentary facies assemblage of the same sedimentary filling sequence stage are obviously different. The filling characteristics of the two aulacogens completely record the geological events related to the breakup of the Colombian supercontinent.

## Introduction

A lot of research on the Cambrian has been carried by many scholars around the world, mainly concentrated in the late Precambrian period. Among them, the oil and gas resources in the Meso-Neoproterozoic strata have been concerned. In the 1970s and 1980s, oil and gas shows were found in Meso-Neoproterozoic stratigraphic in China^1–5^. A set of widely distributed and relatively complete Meso-Neoproterozoic strata is deposited on the crystalline basement of the North China Craton, which is one of the three continents with the best Meso-Neoproterozoic continuous strata in the world^6^. Seismic, drilling and field outcrop data confirm that multiple Aulacogen in Craton were developed in the Meso-Neoproterozoic, and develop multiple sets of source reservoir cap rock combinations that are conducive to the preservation of oil and gas resources. It shows great potential for oil and gas exploration and has attracted the close attention of many scholars^7–9^. In recent years, a lot of research on the development and evolution of the basin has been done and a series of innovative results have been achieved^10–35^. However, there is a lack of systematic summary of its sedimentary characteristics and sedimentary systems, and there is no differential comparison and cause analysis of its sedimentary evolution. Addressing the above problems, The Meso-Neoproterozoic of two typical Aulacogen, Yanliao Aulacogen and Xiong'er Aulacogen, are selected on the north and south sides to clarify the sedimentary evolution characteristics and differences of the typical Meso-Neoproterozoic Aulacogen in the North China. This study also dissects the formation mechanism of differential evolution of typical Aulacogen. It provides sedimentary evidence for the study of the Meso-Neoproterozoic reservoir formation and the evaluation of the exploration field in North China.

## Geologic setting

**Figure 1:**
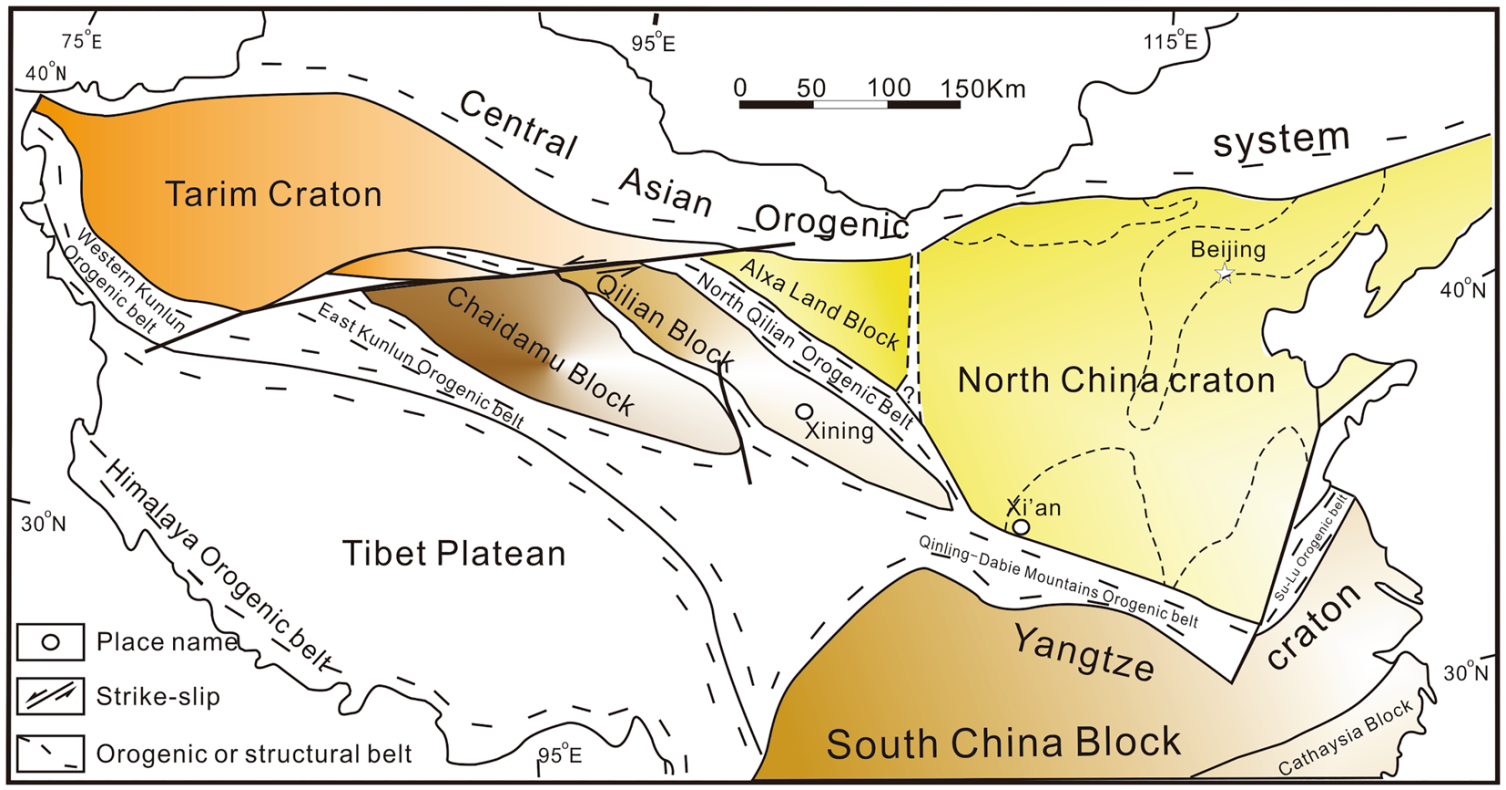
Schematic diagram of Tectonic framework in China (showing the major Precambrian cratons, landmass and Phanerozoic orogenic belt (modified from^44^).

The North China Craton is located in eastern China, recording the earliest ~ 3.8 Ga tectonic history^36^ and multi-block interaction (Fig. 1). The North China Craton has experienced three important geological evolution stages, including the formation of crystalline basement, the development of cap rock and the strong activity of crust. In the formation stage of crystalline basement, a large number of metamorphic rocks and magmatic rocks are exposed on the surface^37–42^. The development stage of cap rock includes the depression sedimentary period of Meso-Neoproterozoic and the quiet crustal activity period of Paleozoic. The thickness of Mesoproterozoic sediments is huge, with a small amount of volcanic rocks, which are mainly deposited in each aulacogen or rift trough. The Meso-Neoproterozoic strata in the Yanliao aulacogen are mainly composed of the Changcheng System, Jixian System, Daijian System and Qingbaikou System. The Xiong ’er aulacogen is mainly composed of Ruyang Group and Luoyu Group. Therefore, there are great differences in stratigraphic development between the two aulacogens. In Paleozoic, the tectonic environment was relatively stable. In quiet crustal activity period of Paleozoic, the lithosphere has obvious thermal thinning characteristics, and the mantle plume has undergone multi-stage evolution, resulting in the formation of the basin range system and the transformation of the Mesozoic tectonic framework^43^.

**Figure 2:**
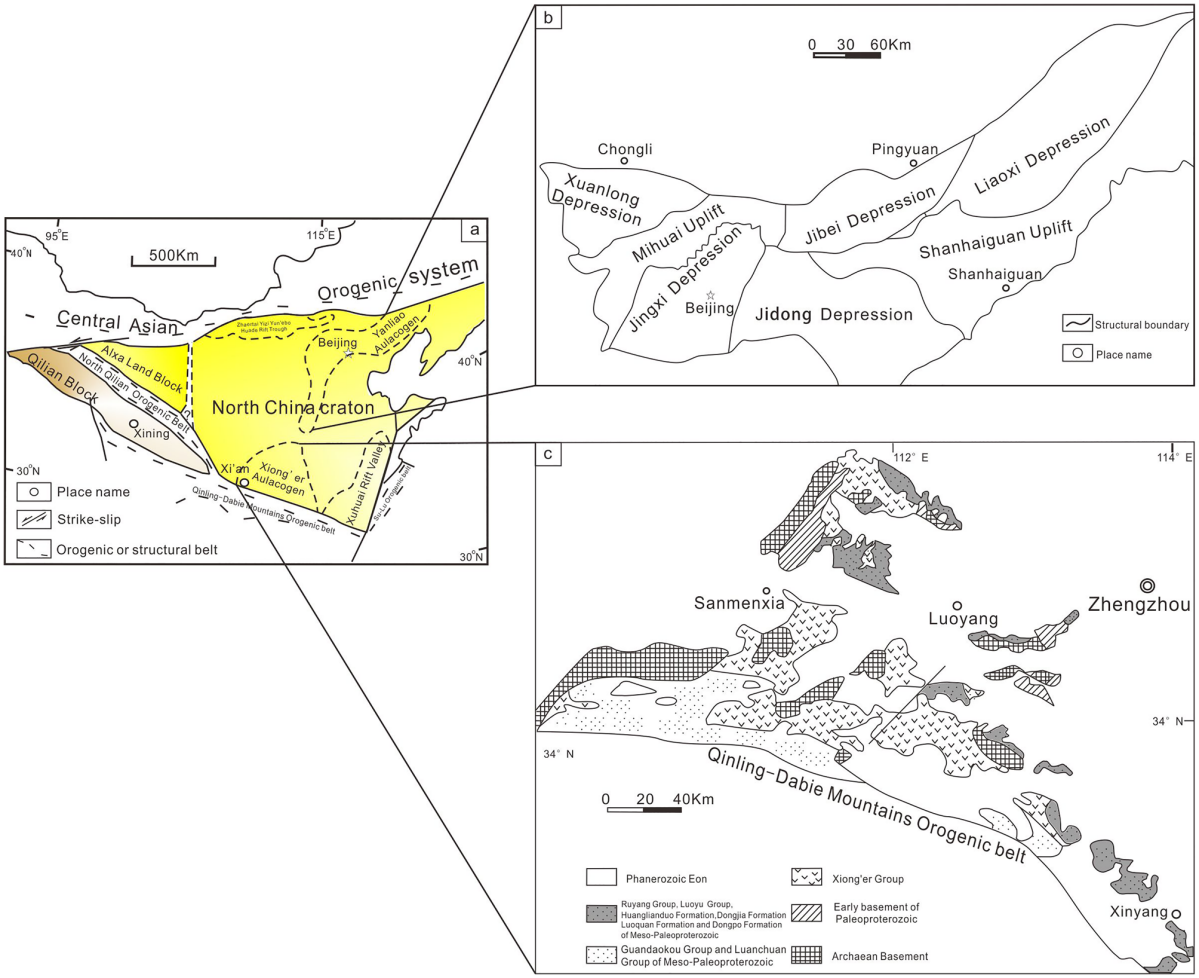
Schematic diagram of Yanliao aulacogen tectonic unit (modified according to^46^). (**a**) Schematic diagram of Tectonic framework in China (modified from^44^); (**b**) Schematic diagram of construction unit of Yanliao aulacogen (modified from^46^); (**c**) Precambrian geological map of the Xiong’ er aulacogen.

The Yanliao aulacogen is located in the northeastern margin of the North China Craton. The overall distribution is in a nearly EW direction, with the eastern section turning into a belt shaped mountain extending in the NE direction (Fig. 2a,b). A set of unmetamorphosed thick (8000 ~ 9000 m) and laterally stable marine carbonate rock intercalated with clastic rock strata was deposited inside. This set of sedimentary rock series is well exposed and widely distributed. From bottom to top, it can be divided into Changcheng System (Changzhougou formation, Chuanlinggou formation, Tuanshanzi formation, and Dahongyu formation) and Jixian System (Gaoyuzhuang formation, Yangzhuang formation, Wumishan formation, Hongshuizhuang formation and Tieling formation) of Mesoproterozoic, Qingbaikou System (Luotuoling Formation and Jingeryu Formation) of Neoproterozoic. The Xiong 'er aulacogen is located in the southern margin of the North China Craton, in the shape of a trifurcated rift^45^ (Fig. 2a,c). After the 'Lüliang' orogenic movement (1.90 ~ 1.85 Ga), the southern margin of the North China Craton was transformed into an extensional tectonic system. Subsequently, a set of thick Meso-Neoproterozoic sediment was deposited in the 'Yu-Jin-Shan' rift zone. Due to the differences in geographical environment, the Meso-Neoproterozoic strata showed different sedimentary characteristics between two aulacogens (Table 1).

**Table 1:** Meso-Neoproterozoic strata correlation table of stratigraphic division in the typical aulacogens of the North China Craton.

Strata		Yanliao aulacogen	Xiong 'er aulacogen			
			Mianchi-Que Mountain	Song Mountain -Ji Mountain	small Qinling Mountains-Luanchuan	Zhongtiao Mountain-Wangwu Mountain
Mesoproterozoic	Changcheng System	Dahongyu Formation	Luoyu Group (Cuizhuang Formation, Sanjiaotang Formation and Luoyukou Formation)	Wufoshan Group (Maanshan Formation, Putaoyu Formation, Luotuopan Formation and Hejiazhai Formation)		Luoyu Group (Cuizhuang Formation, Luoyukou Formation)
		Tuanshanzi Formation	Ruyang Group (Xiaogoubei Formation, Yunmengshan Formation, Baicaoping Formation and Beidajian Formation)	Bingmagou Formation	Gaoshan River Group (Biegaizi Formation, Erdaohe Formation and Chenjiajian Formation)	Ruyang Group (Yunmeng Mountain Formation, Baicaoping Formation, Beidajian Formation)
		Chuanlinggou Formation	Xiong'er Group	Xiong 'er Group	Xiong 'er Group	Xiong 'er Group
		Changzhougou Formation				
	Jixian System	Tieling Formation	Huanglianduo Formation		Guandaokou group (Longjiayuan Formation, Xunjiansi Formation, Duguan Formation and Fengjiawan Formation)	Longjiayuan Formation
		Hongshuizhuang Formation				
		Wumishan Formation				
		Yangzhuang Formation				
		Gaoyuzhuang Formation				
	Pending System	Xiamaling Formation				
Neoproterozoic	Tanian System		Dongpo Formation	Luoquan Formation	Dazhuang Formation	Luoquan Formation
			Luoquan Formation			
	Qingbaikou System	Jingeryu Formation	Dongjia Formation		Luanchuan group (Sanchuan Formation, Nannihu Formation, Meiyaogou Formation, Dahongkou Formation)	

## Materials and methods

The research data is composed of field profiles, cores, and analytical test data in the two aulacogens of Yanliao and Xiong 'er. The focus of this study is based on a large number of outcrops. The division and comparison of the Meso-Neoproterozoic strata in the typical aulacogens in the study area, the types and characteristics of sedimentary facies, and sedimentary filling sequences were analyzed. Based on the study of the Typical section facies, lateral comparison section facies, and sedimentary filling sequences in the two aulacogens of Yanliao and Xiong 'er, the sedimentary evolution difference comparison is carried out, and the response characteristics of geological events are discussed.

## Results

### Types and characteristics of sedimentary facies in aulacogen

Based on the measurement and observation of typical outcrops in the field, and combined with previous research results and regional geological background, the sedimentary facies are identified and divided according to the lithology (color, lithology and structure), sedimentary structure and earth chemistry sigh. The Meso-Neoproterozoic strata in the study area is divided into four sedimentary systems and seven types of sedimentary facies: (1) Marine clastic rock sedimentary system : mainly including barrier coast facies, non-barrier coast facies and shallow shelf facies; (2) Marine carbonate sedimentary system: mainly including carbonate platform facies and reef facies; (3) Marine-continental transitional facies sedimentary system: fan delta facies; (4) Glacier sedimentary system: glacier facies (Table 2). According to its sedimentary characteristics, 15 subfacies and 21 microfacies are further divided.

**Table 2:** Sedimentary facies types and sedimentary characteristics of Meso-Neoproterozoic strata in the study area.

Sedimentary Systems	Facies	Subfacies	Microfacies	Photograph		Characteristics of sedimentary microfacies	Distribution layer	
							Xiong 'er aulacogen	Yanliao aulacogen
Marine clastic rock sedimentary system	Barrier-type coastal facies	Clastic rock tidal flat	Supralittoral zone		Purple red and grayish green mudstone, Yongji section, Shanxi,Baicaoping Formation	Dominated by mudstone, horizontal bedding, mud crack structure, microbial genetic structure and halite pseudocrystal. The grain size probability curve zone of sandstone is mainly composed of suspended components and jumping components	Baicaoping, Beidajian and Changzhougou Formation	Chuanlinggou Formation
			Intertidal zone		Flat gravel, Yongji section, Shanxi,Baicaoping Formation	Characterized by sand-mud interbedding, and developing vein, wavy and lenticular bedding and flat mud gravel. The grain size probability curve is mainly two-stage type, and the jump component is mostly two sub-populations		
			Subtidal zone		Large scale interlaced bedding, Licheng section, Shanxi, Changzhou Gou Formation	Mainly composed of sandstone, large wedge-shaped and plate-shaped cross bedding, pinnate cross bedding and lenticular bedding. The probability curve of sandstone grain size is mainly presented as two sections, which are composed of three or two grain size sub-populations		
		Barrier bar	Barrier sand		Barrier Dam and Lagoon, Licheng Section, Shanxi,Chuanlinggou Formation	Dominated by sandstone, bidirectional cross bedding, migration bedding, trough cross bedding and lenticular bedding. The tidal channel sandstone is dominated by jumping and suspended components, and the barrier dam sandstone is dominated by jumping components	Chuanlinggou Formation ,Changzhougou Formation	Chuanlinggou Formation
		Lagoon	Lagoon mud		Black shale, Yongji section, Shanxi, Beidajian Formation	Mudstone, silty mudstone, horizontal bedding	Beidajian, Zhaojiazhuang and Changzhougou Formation	–
	No-barrier coast facies	Backshore	Backshore sand		Low angle interlaced bedding, Yuntai Mountain profile, Henan, Yunmengshan Formation	Fine sandstone, parallel bedding, low angle cross bedding and wavelet mark bedding. The grain size probability curve zone of sandstone is mainly two-stage or three-stage	Yunmengshan, Ma 'an Mountain Formation	–
		Foreshore	Foreshore sand (gravel)		Flushing cross bedding, Yuntai Mountain profile, Henan,Yunmengshan Formation	Medium-fine sandstones, flushed cross-bedding, wavy ripples, parallel bedding, low-angle cross-bedding, glauconite-bearing and microbial genetic structures. The probability curve of sandstone grain size is mainly presented to two-stage	Yunmengshan, Baicaoping, Beidajian, Sanjiaotang, Dongjia, Ma 'an Mountain, Erdaohe River and Sanchuan Formation	Changzhougou, Xiamaling and Luotuoling Formation
		Nearshore	Nearshore sand		Interbedded shale and sandstone, Zhaojiashan section, Hebei, Longshan Formation	Fine sandstone, occasionally intercalated mudstone thin layer, seeing ripples, parallel bedding, sandstone grain size probability curve zone is mainly two-stage	Yunmengshan, Baicaoping, Beidajian, Sanjiaotang, Dongjia, Ma 'an Mountain , Erdaohe River and Sanchuan Formation	Changzhougou, Xiamaling and Luotuoling Formation
	Shallow marine shelf facies	Transition zone	Transition zone sand and mud		Interlayer of siltstone and grayish green shale, Beizhangzi section in Hebei Province, Xiamaling Formation	Gray green shale, dark shale and siltstone interbedded, horizontal bedding	Cuizhuang and Luoquan Formation	Changzhougou and Xiamaling Formation
		Offshore shelf	Offshore shelf mud		Black mud shale, Yongji section, Shanxi, Cuizhuang Formation	Gray black, dark gray shale, black siliceous shale, horizontal bedding	Cuizhuang and Luoquan Formation	Xiamaling Formation
Marine carbonate sedimentary system	Carbonate platform facies	Carbonate tidal flat	Supralittoral zone		Dry crack structure, Kuancheng section in Hebei, Wumishan Formation	Mud crystal dolomite, horizontal laminae, drying split, birds-eye structure, contraction joints, asymmetric ripples and so on	The upper part of Beidajian, Luoyukou, Huanglianduo and Longjiayuan Formation	Jingeryu , Tieling, Hongshuizhuang, Wumishan, Yangzhuang, Gaoyuzhuang, upper part of Dahongyu, Tuanshanzi and upper part of Chuanlinggou Formation
			Intertidal zone		Hemispherical laminated stone, Yongji section, Shanxi, Longjiayuan Formation	Powder crystal dolomite, sandy dolomite, columnar stromatolites, cross bedding		
			Subtidal zone		Core shaped stone, Kuancheng section, Hebei, Wumishan Formation	Fine-grained dolomite, oolitic dolomite, internal clastic dolomite, seeing large argillaceous bands		
		Bay-lagoon	Bay-lagoon mud		Black gray shale, Hebei Beizhangzi section, Honghongzhuang Formation	Dark mud shale is intercalated with thin layer of micritic (argillaceous) dolomite, horizontal bedding, containing iron-manganese nodules and pyrite	/	Gaoyuzhuang and Hongshuizhuang Formation
	Bioherm facies	Build-up- stromatolite hill	Stromatolite dolomite		Barrier deposit cohesive rock, Zhaojiashan section, Hebei, Gaogaozhuang Formation	Light gray massive stromatolites or algal dolomite bioherm	/	Ninth and tenth sections of Gaoyuzhuang Formation
Marine-continental transitional sedimentary system	Fan delta	Fan-delta plain	Distributary river channel		Developed plate-like cross bedding, Henan Daimeishan section, Xiaogoubei Formation	Conglomerate, (gravel-bearing) coarse sandstone, seeing cross bedding, erosion surface	Xiaogoubei and Bingmagou Formation	–
			Interdistributary bay		Grayish yellow mud shale, Wan'anshan section, Henan, Bingmagou Formation	Argillaceous sandstone, siltstone, shale, bedding is not well developed		
		Fan delta front	Underwater distributary river channel		Cross bedding and erosion structures, Wan'an Mountain section, Henan, Bingmagou Formation	Pebbled sandstone, coarse sandstone, developing cross bedding, erosion surface, single sand body is lenticular, with normal-graded	Xiaogoubei and Bingmagou Formation	–
			Interdistributary bay		Purple red sandy shale interbedded with gray green sandy mudstone, Wan'anshan section, Henan, Bingmagou Formation	Silty mudstone and fine- silty sandstone interbedded, seeing horizontal bedding		
Glacier sedimentary system	Glacier	Glacial moraine	Moraine rocks		Moraine rocks, Lushan Section, Henan, Luoquan Formation	Sandy conglomerate, mixed composition, poor sorting and rounding, glacier scratches on the surface of gravel	Luoquan Formation	–
		Glaciomarine	Shallow glaciomarine		Grey green shale interbedded with yellow yellow siltstone, Lushan Section, Henan, Luoquan Formation	Mainly argillaceous sediment, with more fine-grained materials, showing rhythmic layers, often showing symbiosis with laminated heterogeneous conglomerate	Luoquan Formation	–

### Characteristics of sedimentary filling sequence

#### Sedimentary filling sequence of Meso-Neoproterozoic in Yanliao aulacogen

**Figure 3:**
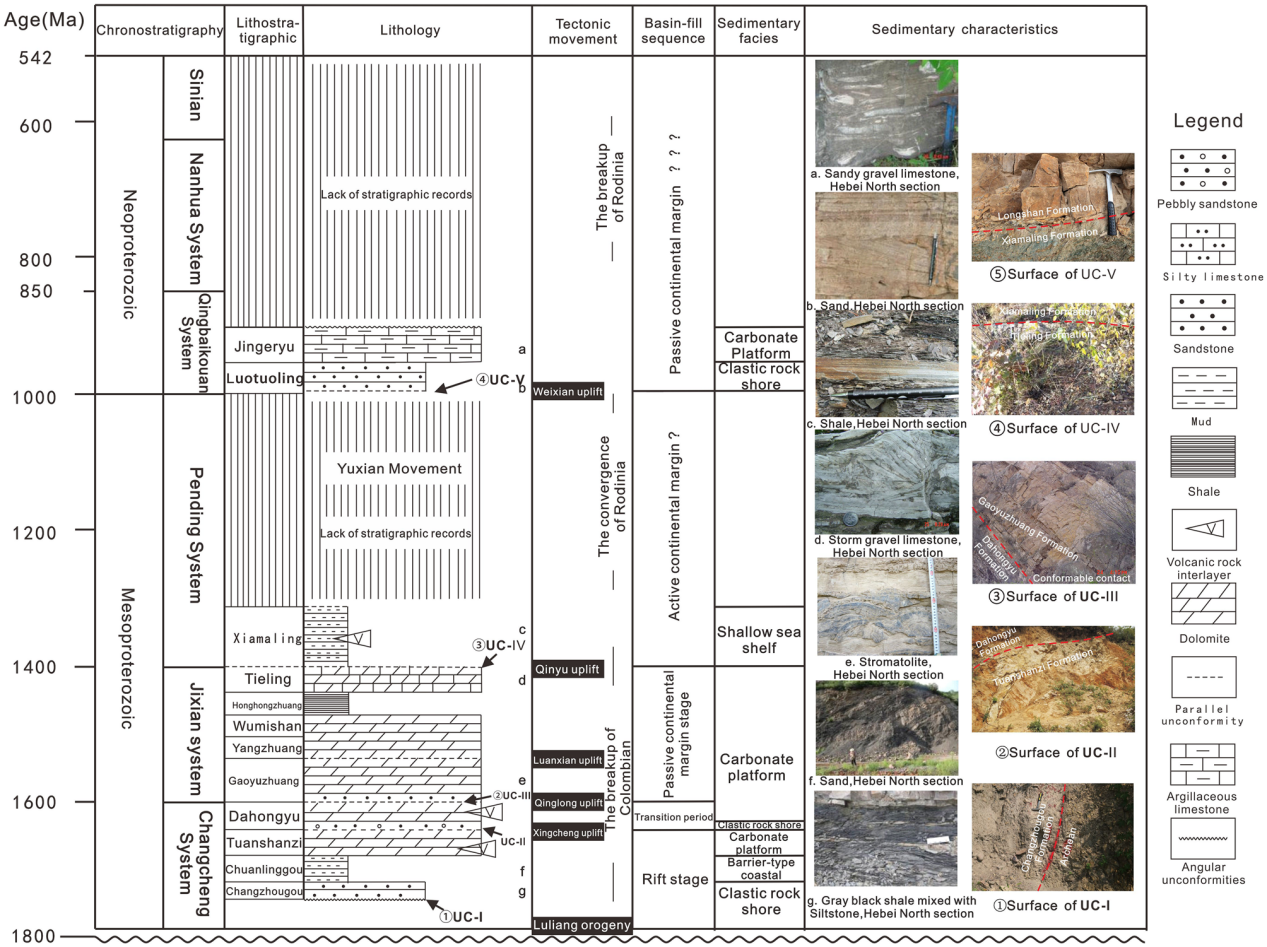
Sedimentary filling sequence diagram of Meso-Neoproterozoic in Yanliao aulacogen.

On the basis of observation and analysis of the two backbone sections of Jibei Depression and Xuanlong Depression in the Yanliao aulacogen, and in combination with regional stratigraphic and structural background data^47–49^, it is believed that the Yanliao Aulacogen experienced four stages in the Mesoproterozoic and one stage in Neoproterozoic. The four stages of Mesoproterozoic are continental rift deposition, transformation from continental rift to passive continental margin, passive continental margin and active continental margin. The formation of the Neoproterozoic Qingbaikou System (starting from the Luotuoling Formation) is the result of a new continental extension, which is a passive continental margin stage (Fig. 3). Five sedimentary sequences are identified in the study area.

(1) Sequence I includes Changzhougou Formation-Tuanshanzi Formation, which was deposited in continental rift stage. It is between the unconformable surface UC-I (Fig. 3①) and UC-II (Fig. 3②)^50^ of the Yanliao aulacogen basement. Among them, the unconformity surface UC-I is the angular unconformity between the sandy conglomerate at the bottom of the Changzhou Formation and the underlying Archean Metamorphic rock series, which is the product of the Luliang movement. The Changzhougou Formation is mainly a set of clastic rock deposits dominated by quartz sandstone with parallel and staggered layers. At the bottom, there are laterally discontinuous gravels, and the overall sedimentary environment is Clastic rock shore-shallow sea-shelf Clastic rock shore (Fig. 3g). The lithology of the Chuanlinggou Formation is dark horizontal layered mudstone, with fine-grained sandstone in the form of lenses or normal-graded bedding. It is an anoxic barrier coastal sedimentary environment (Fig. 3f). The Tuanshanzi Formation develops a set of Carbonate rock intercalated with fine Clastic rock deposits, and the sedimentary environment becomes Carbonate rock tidal flat. The distribution range of them is relatively limited, and their sedimentary thickness varies greatly^51–53^.

(2) Sequence II includes the Dahongyu Formation, representing the transformation sedimentary from continental rift to passive continental margin. It was deposited on the top of the Great Wall System in the Yanliao Aulacogen, and generally distributed in the NE direction, between the unconformity surface UC-II and UC-III (Fig. 3③). Unconformity surface UC-II is an overlap unconformity between Dahongyu Formation and old strata. It is mainly a set of clastic rocks intercalated with volcanic rocks and dolomite deposits. In the early sedimentary period of Dahongyu Formation, it is mainly a set of clastic rocks with volcanic rocks. The grain size at the bottom is coarse and the hydrodynamic force is strong, indicating the beginning of basin expansion, extensive transgression and thermal subsidence. Upward, it is mainly composed of medium-fine grained sediments, with dolomite and a large number of volcanic rock interlayers. It is a foreshore sedimentary environment of no-barrier coast facies. The upper part is interbedded with sandy dolomite and argillaceous dolomite, which is a supralittoral-intertidal zone sedimentary environment of carbonate rock, indicating that the crust is relatively rising and sea level is falling.

(3) Sequence III includes Gaoyuzhuang Formation-Tieling Formation, which represents passive continental margin deposits, with a sedimentary system dominated by tidal flat and bay-lagoon sedimentary environments. It is between unconformity surface UC-III and UC-IV (Fig. 3④). Unconformity surface UC-III is the unconformity between Gaoyuzhuang Formation and old strata. During the sedimentary period of Gaoyuzhuang Formation, the whole sedimentary environment is carbonate tidal flat, containing a lot of stromatolites, with siliceous or algae ash clumps and nodules (Fig. 3e). The Yangzhuang Formation is characterized by purple-red silty argillaceous dolomite, which is generally intertidal-supralittoral zone deposit of carbonate rock tidal flat. The sea level gradually rises from bottom to top. The Wumishan period inherits the late trend of Yangzhuang Formation, with typical sedimentary rhythm layer. During the deposition period of the Hongshuizhuang Formation, the sedimentary environment was a bay-lagoon sedimentary environment. The sediments were mainly gray-black shale, and the sea level gradually decreased. During the sedimentary period of Tieling Formation, the sediments were dominated by limestone and dolomite limestone, with a large number of stromatolites. With the rise of sea level, the environment changes from intertidal-supralittoral zone to subtidal zone (Fig. 3d).

(4) Sequence IV includes Xiamaling Formation, which represents active continental margin deposits. It is between unconformity surface UC-IV and UC-V. The Xiamaling Formation is mainly a set of shale, mudstone, occasionally intercalated with marl, argillaceous dolomite and sandstone, and a large number of diabase sheets (Fig. 3c). It shows that the Yanliao aulacogen was in an obvious extension state during this period.

(5) Sequence V includes the Luotuoling Formation-Jing 'eryu Formation, which is a passive continental margin deposit. It is between the unconformity surface UC-V and UC-VI. The unconformity surface UC-V is a parallel unconformity between the Luotuoling Formation and the Xiamaling Formation (Fig. 3⑤), and even a micro-angular unconformity. The Luotuoling Formation is mainly a set of containing glauconite quartzarenite and silty shale, which is a clastic rock littoral-shallow marine shelf environment (Fig. 3b). The Jing 'eryu Formation is mainly a set of marine carbonate subtidal deposits (Fig. 3a). The sedimentary range of the two should be wider than the present residual part, reflecting a relatively stable environment of Epicontinental sea.

#### Sedimentary filling sequence of Middle-Neoproterozoic in Xiong 'er aulacogen

**Figure 4:**
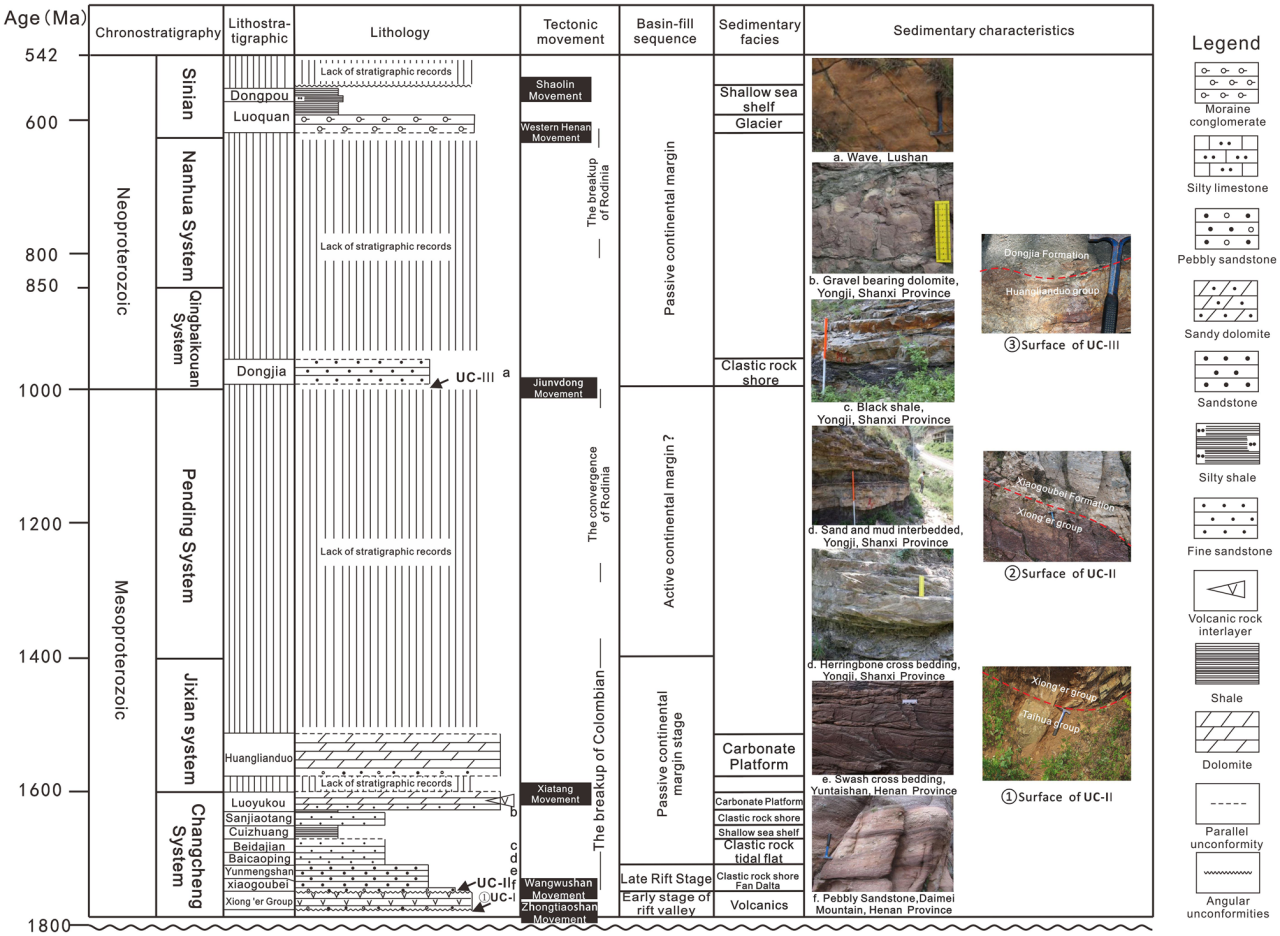
Sedimentary filling sequence diagram of Meso-Neoproterozoic in Xiong 'er aulacogen.

On the basis of the lithological characteristics, contact relationship, vertical sequence, and structural background of the Meso-Neoproterozoic strata in the Xionger Aulacogen field section, and in combination with the sedimentary characteristics of three typical backbone sections and one horizontal correlation section^51–53^, it is considered that the Meso-Neoproterozoic strata in Xiong 'er aulacogen have experienced three stages: early continental rift, late continental rift and passive continental margin (Fig. 4). Four sedimentary sequences are identified in the study area.

(1) Sequence I includes the whole Xiong 'er Group, which is located at the bottom of Changcheng System of Xiong 'er Aulacogen. It is between the unconformity surface UC-I and UC-II. Unconformity surface UC-I is angular unconformity contact between Xiong 'er group and underlying Archean Taihua Group Gneiss basement or paleoproterozoic Shuangfang Group biotite quartz schist (Fig. 4①)^54^. Sequence I is a set of volcanic lava with a small amount of sedimentary rocks or pyroclastic rock depositions (Xiong 'er Group). From bottom to top, alluvial fan-fan delta deposits of Dagushi Formation are successively developed. The Xushan Formation, Jidanping Formation and Majiahe Formation are volcanic rock deposits. And upward, the sedimentary rock interlayer thickens and increases. It indicates that this is the early stage of the rift and the transgression has reached the Xiong 'er aulacogen.

(2) Sequence II mainly includes the Xiaogoubei Formation and the Yunmengshan Formation. It develops above the unconformable surface UC-II (Fig. 4②) and below the Baicaoping Formation. Although the Yunmengshan Formation and the overlying Baicaoping Formation in the study area are mostly in integrated contact, in the Zhongtiao Mountains area, the Baicaoping Formation overlaps and unconformably above the Xionger Group. The Xiaogoubei Formation and Yunmengshan Formation are characterized by a set of coarse clastic sediments, among which the Xiaogoubei Formation is fan delta of marine-continental transitional sedimentary system (Fig. 4e,f). As sea level continues to rise, a coastal sedimentary environment is formed during the transgression of the Yunmengshan Formation. The whole sequence II is characterized by the late rift deposition in the transgression environment.

(3) Sequence III includes the Baicaoping Formation-Huanglianduo Formation, which developed under the unconformity surface UC-III. The unconformity surface UC-III is a parallel unconformity between the Huanglianduo Formation and the Dongjia Formation (Fig. 4③). During the sedimentary period of Baicaoping Formation, a set of mud shale with a small amount of sandstone deposits was formed (Fig. 4d). The water body of the Cuizhuang Formation continues to deepen, gradually evolving from the early nearshore-foreshore sedimentary environment to the late shallow sea shelf sedimentation. During the sedimentary period of the Sanjiaotang Formation, a set of fine-grained quartz sandstone deposits was formed, belonging to coastal sedimentation (Fig. 4b). During the sedimentary period of Luoyukou Formation, the sediments changed from clastic rocks to carbonate rocks, and the sedimentary environment changed from transitional zone to Carbonate rock tidal flat. The sedimentary environment of the whole sequence is mainly clastic rock shore-shallow sea shelf-carbonate platform, representing passive continental margin deposits.

(4) A large number of strata are missing in sequence IV, which is between the unconformity surface UC-III and UC-IV. It includes the Dongjia Formation-Dongpo Formation. The unconformity surface UC-IV is the parallel unconformity between the Dongpo Formation and the overlying Cambrian Xinji Formation. The early and middle stages of the Qingbaikou period are mainly clastic rock deposits, and its gravels are mainly composed of dolomite, banded siliceous rock, vein quartz, etc. (Fig. 4a), which reflects the sedimentary environment of clastic rock littoral. The Luoquan Formation of Sinian System is a typical set of glacier deposits. At the end of Sinian, the Dongpo Formation experienced another large-scale transgression, with fine sandstone at the bottom and shale mixed with Siltstone at the top, which is a coastal-shallow sea shelf sedimentary environment. Sequence IV developed clastic rock littoral-carbonate platform—glacier-clastic rock littoral-shallow marine shelf sedimentary environment, which represents passive continental margin deposits.

## Discussion

**Figure 5:**
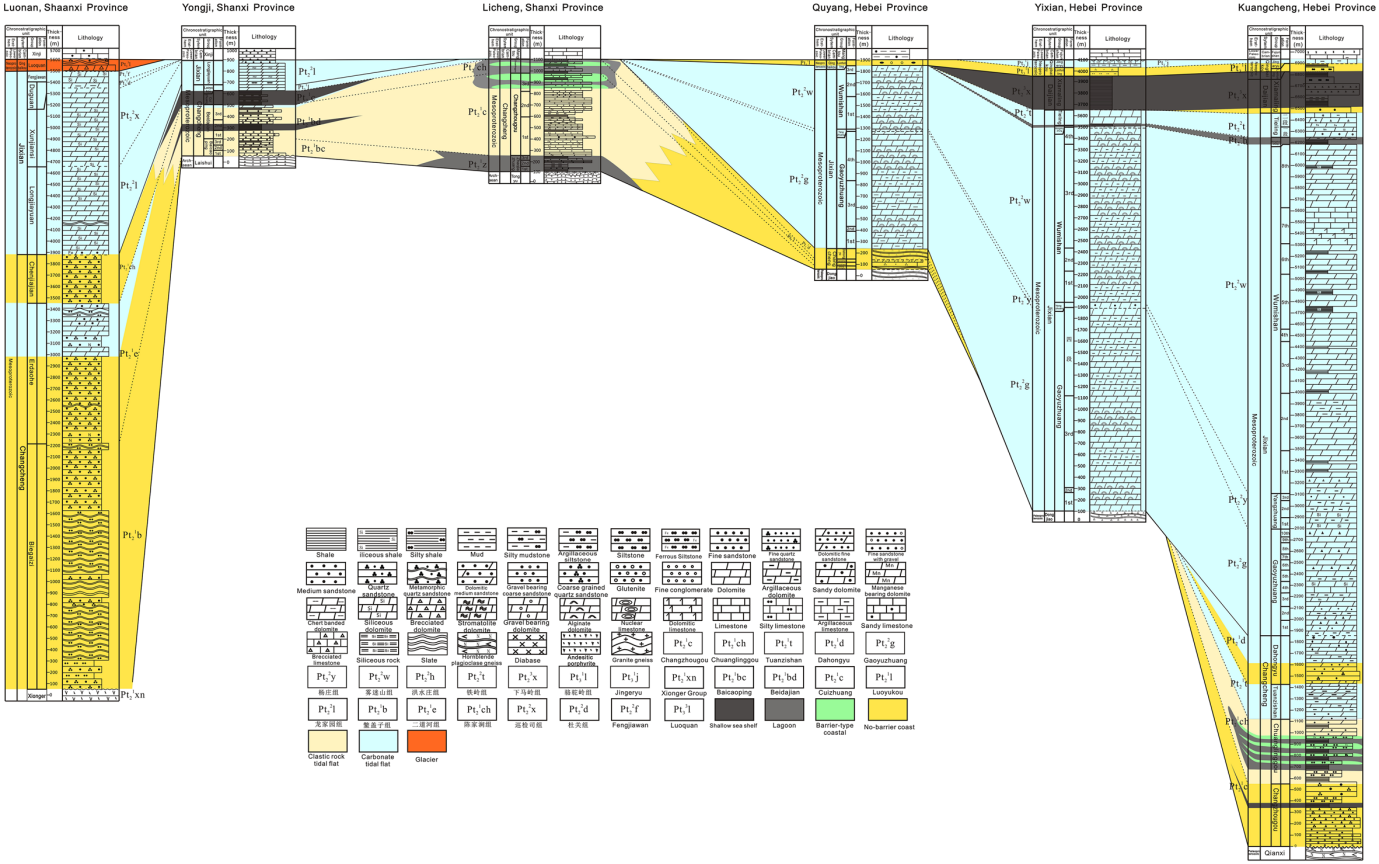
The sedimentary facies profile of the Meso-Neoproterozoic in Luonan, Shanxi-Yongji, Shanxi-Licheng, Shanxi-Quyang, Hebei-Yixian, Hebei-Kuancheng, Hebei.

In this study, the horizontal comparison section of sedimentary facies is selected for comparative analysis, which is Shanxi Luonan-Shanxi Yongji-Shanxi Licheng-Hebei Quyang-Hebei Yixian-Hebei Kuancheng (Fig. 5). In combination with the distribution characteristics of the Meso-Neoproterozoic strata in each Aulacogen, the sedimentary filling sequence has obvious differences, mainly in three aspects:

### Comparison of the formation time of each sedimentary filling sequence stage of different aulacogens

The rifting of the Yanliao Aulacogen started in the Changzhougou period of the Changcheng period in the Mesoproterozoic (~ 1650 Ma). The continental rift stage occurred in the Changzhougou-Tuanshanzi period of the Changcheng period. The Dahongyu period of the Changcheng period is the transformation stage from continental rift to passive continental margin. The passive continental margin stage occurred in the Gaoyuzhuang-Tielingian Jixianian period. The Daijian period is a active continental margin stage. The Neoproterozoic Qingbaikouan is the second passive continental margin stage.

The rifting of Xiong 'er aulacogen began in the early Changcheng period of Mesoproterozoic (~ 1780 Ma). The sedimentary period of the Xiong'er Group during the Changcheng period is the early stage of the continental rift. The Xiaogoubei-Yunmengshan period of the Changcheng period is the late stage of the continental rift. From the Baicaoping period of the Changcheng period to Jixianian period, it is in the passive continental margin stage. The sedimentary strata of the Daijian period are missing, speculating that it is the active continental margin stage. Qingbaikou period is the second passive continental margin stage.

### Comparison of characteristics of each sedimentary filling sequence of different aulacogens

The continental rift stage: The lower part of Yanliao aulacogen (the Changzhougou Formation) is mainly a set of clastic rock deposits, and the conglomerate layer is developed at the bottom. The Chuanlinggou Formation is dark horizontal layered mudstone. The Tuanshanzi Formation is a set of carbonate rocks with fine clastic rocks, and volcanic rocks are widely developed locally. The whole is clastic rock shore-anoxic barrier coast sedimentary-carbonate tidal flat sedimentary environment. During early stage of rifting in the Xionger Aulacogen depression, a set of ultra thick andesitic volcanic rocks of Xiong 'er Group were developed in Xiong 'er Formation. A set of coarse clastic sedimentary rocks developed in Xiaogoubei Formation and Yunmengshan Formation during late stage of rifting. The whole is fan delta-clastic rock shore sedimentary environment.

The transition period (the transformation from continental rift to passive continental margin): It is obvious only in the Yanliao aulacogen, which develops a set of clastic rock with volcanic rock deposition. In the late sedimentary period, the sediments were mainly dolomite. In the late stage, although the rifting and volcanism continued, its intensity decreased significantly. The environment changes from no-barrier coast facies to carbonate rock platform facies. However, no developmental characteristics were found about this stage in the Xiong 'er aulacogen.

The passive continental margin stage: the Jixian System in Yanliao aulacogen is mainly composed of thick carbonate rocks and mudstones, and the overall sedimentary environment is carbonate tidal flat-shallow marine shelf-carbonate tidal flat. In the Xiong 'er aulacogen, the early stage (Baicaoping Formation-Sanjiaotang Formation) develop a set of fine clastic rock deposits dominated by quartz sandstone, siltstone and shale. In the late stage (Luoyukou Formation and Huanglianduo Formation), a set of carbonate deposits was developed. The whole is clastic rock littoral-shallow marine shelf-carbonate platform sedimentary environment.

The active continental margin stage: The Yanliao aulacogen is mainly a set of shale and mudstone, occasionally intercalated with marl, argillaceous dolomite and sandstone. And a large number of diabase sheets appeared. It is a shallow marine shelf sedimentary environment. In Xiong 'er aulacogen, it is speculated that the sedimentary stratum is missing due to Continental collision and Tectonic uplift in this stage.

The second passive continental margin stage: The Yanliao aulacogen is mainly composed of clastic rocks and carbonate rocks, which developed in clastic rock littoral-carbonate tidal flat sedimentary environment. In Xiong 'er aulacogen, the Dongjia Formation of Qingbaikou System is a set of terrigenous clastic-carbonate rock formation. After that, there was one set of stratigraphic deposits only in Yuku Formation of the Nanhua period in the Xiaoqinling-Luanchuan stratigraphic area, which is mainly a set of metamorphic rocks constructed by volcanic rocks and carbonate rocks. In the late stage (Luoquan Formation and Dongpo Formation in Sinian period), a set of moraine glutenite, (gravel-bearing) fine sandstone, siltstone and shale were developed^51,52^. The whole is clastic rock littoral-carbonate platform-glacier-clastic rock littoral -shallow marine shelf sedimentary environment.

### Response characteristics of sedimentary filling to important geological events

**Figure 6:**
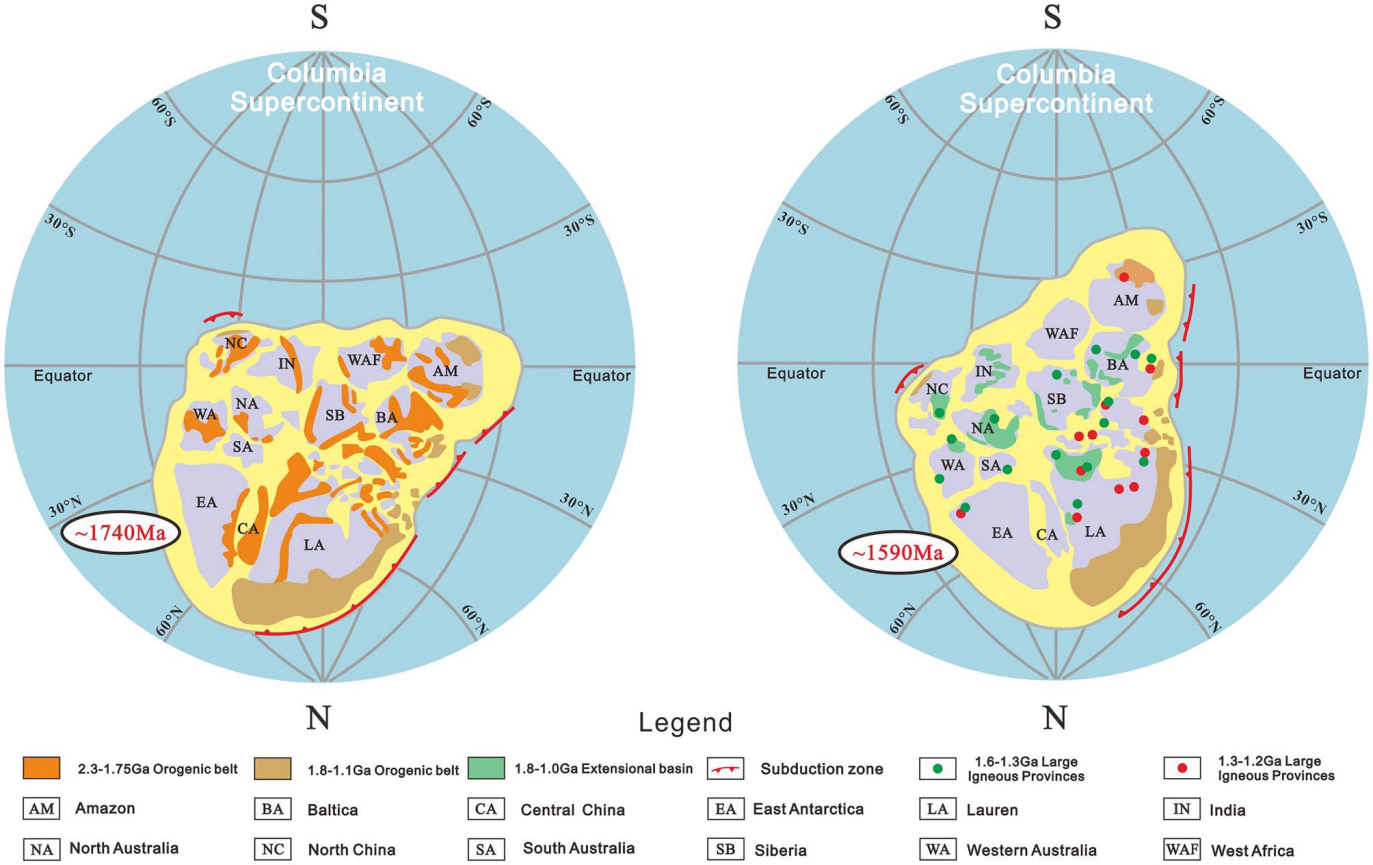
Supercontinental configuration and paleogeographic location of Colombia (modified from^59^).

The deposition process of the North China Craton in the middle Proterozoic (about 1.8 ~ 1.35 Ga) completely recorded the breakup process of the Columbia supercontinent^55^. Following the convergence and formation of the Colombian Supercontinent, the North China Craton experienced a series of convergence, collision, amalgamation and unified crystallization in the Paleoproterozoic, and the North China Craton basement formed^56–58^. Subsequently, the North China Craton was incorporated into the Colombian supercontinent and remained relatively stable for a long time (Fig. 6)^59^. This series of tectonic movements make obvious differences in sedimentary filling in different aulacogens.

#### The Yanliao aulacogen

**Figure 7:**
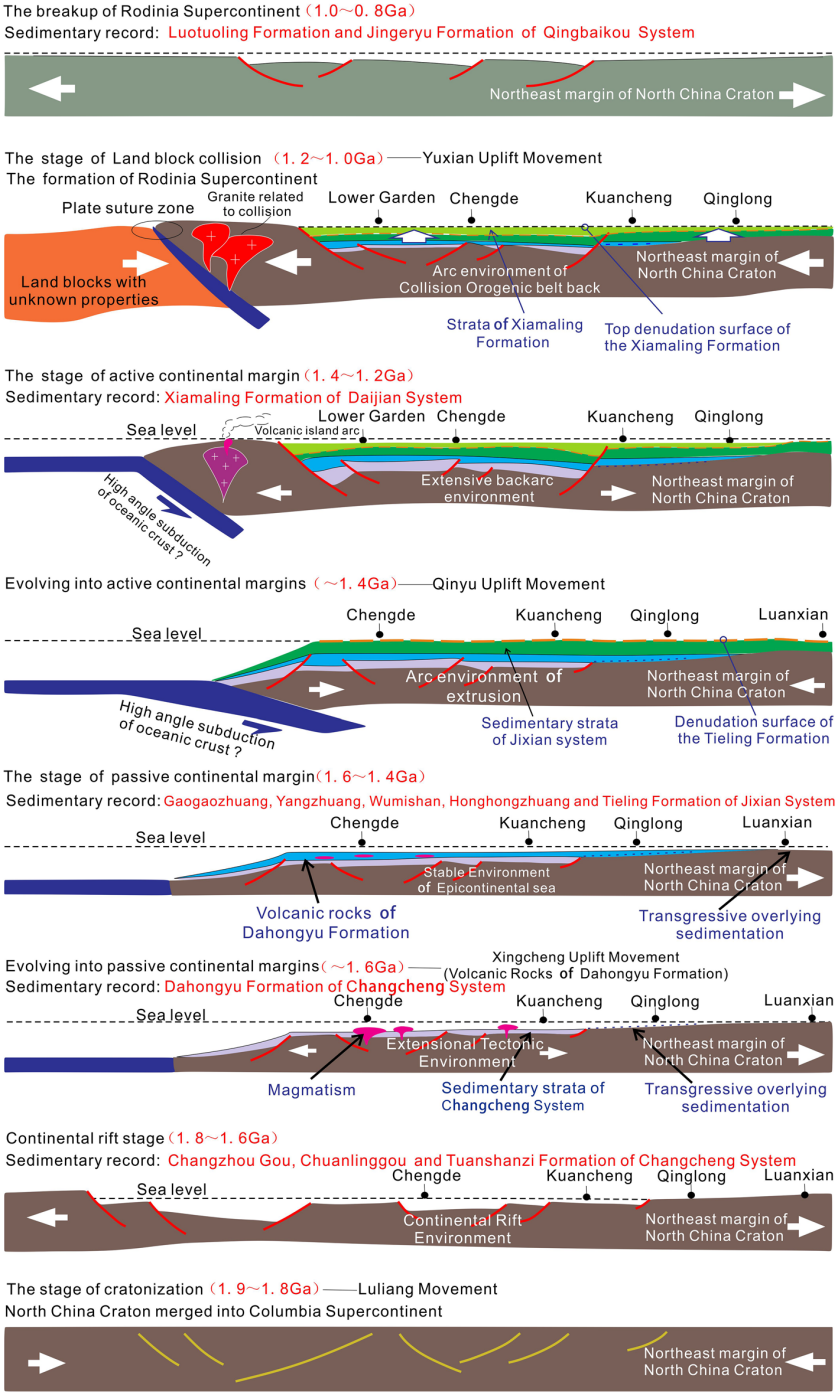
Meso-Neoproterozoic tectonic evolution diagram of Yanliao aulacogen^60^.

The Changcheng period of Yanliao aulacogen is dominated by a large set of terrigenous clastic rock deposits, which are unconformable on the underlying Archean Metamorphic rock series at an angle, with a sedimentary thickness of 2670 m. It indicates that the rifting is obvious. The stratigraphic distribution characteristics show that the basin has the characteristics of continental rift and aulacogen. The Changzhougou-Tuanshanzi period is the continental rift stage. With the eruption of volcanic rocks, a set of clastic rock with volcanic sedimentary formation is developed in Dahongyu Formation. As the intensity of activity decreases, the basin transforms from a continental rift to a passive continental margin during the Dahongyu period (Fig. 7). Dahongyu Formation is in parallel unconformity with the underlying Tuanshanzi Formation, or in direct overlap unconformity with Archean crystalline rocks. The overlap unconformity is a geological response record of the ‘Xingcheng Uplift’ tectonic event (Fig. 7).

The tectonic environment of Jixian period tends to be stable and has turned into a passive continental margin stage. A set of extremely thick carbonate rocks with muddy is developed, which is dominated by a large area of carbonate platform sedimentary environment (Fig. 7). The regional discordant contact at the bottom of the Jixian system is a geological response record of the ‘Qinglong Uplift’ tectonic event in the area. In the late Gaoyuzhuang period, the Taihang highland gradually expanded and the sea level decreased relatively. The sea water retreats northward, resulting in a reduction in the area of land surface sea basins. As a result, sedimentary hiatus occurred in Gaoyuzhuang Formation and Yangzhuang Formation at the edge of the basin, which is the geological response record of the 'Luanxian Uplift' tectonic event in this area. At the end of the deposition of the Tieling Formation of the Pending Period, the crust uplifts as a whole, and the area of the epicontinental sea basin is further reduced. As a result, it led to the uplift and erosion of the Tieling Formation, forming a large area of ancient weathering crust. Subsequently, a set of shale intercalated with argillaceous carbonate rock or fine sandstone deposits were deposited in the upper part. During the sedimentary period of the Xiamaling Formation, the Yanliao aulacogen is in an obvious extensional state and it belongs to active continental margin deposition (Fig. 7). Due to the overall uplift of the crust, the distributions of the Xiamaling Formation in this area are small, which is parallel to the Tieling Formation. It is the geological response record of the 'Qinyu Uplift' tectonic event. After that, the crust of North China uplifted and squeezed.

It led to a long period of denudation (erosion) and planation after the deposition of Xiamaling Formation and before the deposition of Luotuoling Formation. It leads to the loss of strata, which is the geological response record of the 'Yuxian Uplift' tectonic event in this area. In Qingbaikou period, the North China Block began to subside again, and the Qingbaikou System is mainly composed of clastic rocks and carbonate rocks, which reflects a relatively stable epicontinental sea structure-sedimentary environment and belongs to passive continental margin deposition (Fig. 7).

#### The Xiong 'er aulacogen

**Figure 8:**
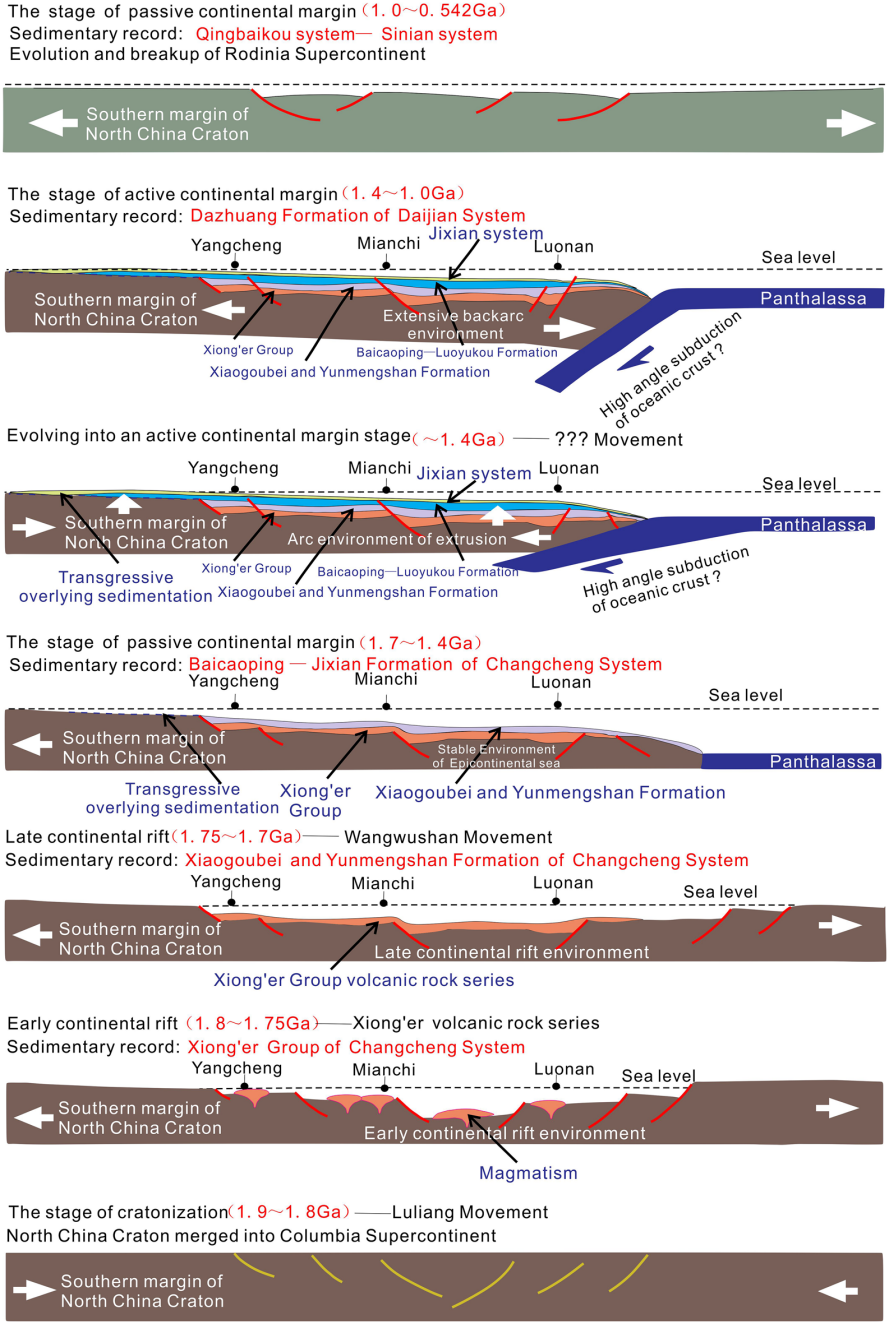
The tectonic evolution diagram of Xiong 'er aulacogen in Meso-Neoproterozoic^60^.

The development of the Xiong 'er aulacogen is also considered to be a geological response record of the North China Craton in the Columbia supercontinent breakup event. At early stage of the Xiong 'er-Yunmengshan Period of Changcheng Period, the southern margin of the North China Craton experienced a short period of initial crustal cracking, with rapid uplift of the mantle plume, intense stretching and thinning of the Lithosphere, and eruption of a large amount of magmatic materials. A set of thick volcanic lava sediments that deposited, which is in angular unconformable contact with the underlying strata and is the product of the ‘Zhongtiao Mountains Movement’ in this area (Fig. 8).

Late continental rift deposition, The coarse debris deposits (Xiaogoubei Formation and Yunmengshan Formation) with a thickness of over one kilometer have been deposited, forming in a fan delta coastal sedimentary environment (Fig. 8). It is in angular unconformable contact with the underlying Xiong 'er Group, which is a geological response record of the ‘Mount Wangwushan Movement’ tectonic event in this area.

With the continuation of the breakup of the Columbia Supercontinent and the extrusion between continents, the tectonic environment became stable during the Baicaoping-Jixian period of the Changcheng Period, and mainly developed clastic rock shore-shallow sea shelf-carbonate rock platform sedimentary environments. Subsequently, the rifting ended and the whole was transformed into passive continental margin deposition. In the early sedimentary stage of the passive continental margin (Baicaoping Formation-Sanjiaotang Formation), a set of quartz sandstone, siltstone and shale has developed. In the late passive continental margin (Luoyukou Formation-Huanglianduo Formation), it is epicontinental sea and sediments mainly are carbonate rocks.

During the Daijian period of Mesoproterozoic, the North China Craton was further uplifted, resulting in extensive loss of Daijian System strata and further narrowing of the sedimentary range of Xiong'er Aulacogen. Only developed the Dazhuang Formation of in the Luonan region of Shanxi Province (Fig. 8). Therefore, this period in the Xiong 'er aulacogen is considered to be the active continental margin stage.

The North China Craton experienced the evolution and breakup of the Rodinia supercontinent in the Neoproterozoic. During the Qingbaikou-Sinian Period, the Xiong 'er aulacogen was a passive continental margin sedimentary stage (Fig. 8). In the Mianchi-Queshan stratigraphic area, it occurred a small-scale transgression during the Dongjia Period. The sedimentary environment changed from clastic rock barrier-free coast to carbonate tidal flat environment. In Xiaoqinling-Luanchuan area, the Qingbaikou System is a set of terrigenous clastic rock-carbonate rock formation. In Songji area, regional tectonic movements led to crustal uplift and loss of strata during the Jixian and Qingbaikou periods. During the Nanhua period, it is only developed the Yuku Formation in the Xiaoqinling-Luanchuan area. It mainly develops a set of carbonate rock formation, which deposit in carbonate platform facies. In the south of North China, an event of the Luoquan Glacial Age occurred, and the Luoquan period entered into the glacial stage. A set of glacial tillite is mainly developed, which is the geological response record of the 'Precambrian Great Ice Age' in the world. At the end of the Sinian Period, the North China plate was uplifted, and the ancient land area was further increased. Due to climate warming and glacier melting, a large-scale transgression occurred. The Dongpo Formation was deposited in clastic rock littoral-shallow marine shelf sedimentary environment.

## Conclusions

1. Guided by the theory of sedimentology, through a large number of outcrops measurement and observation, core observation and description, the sedimentary facies division marks such as lithology, structural marks and sedimentary structure marks were analyzed, and the division schemes of sedimentary facies, subfacies and microfacies were established. The sedimentary facies types of the Meso-Neoproterozoic in the study area were divided into four sedimentary systems and seven sedimentary facies types. (1) Marine clastic rock sedimentary system : mainly including barrier coastal facies, non-barrier coastal facies and shallow shelf facies; (2) Marine carbonate sedimentary system : mainly including carbonate platform facies and reef facies; (3) glacier sedimentary system : glacial facies ; 4)Marine-continental transitional facies sedimentary system : fan delta facies ; according to its sedimentary characteristics, 15 subfacies and 21 microfacies are divided. Among them, the non-barrier coastal facies, carbonate platform facies and shallow shelf facies are the main sedimentary facies types of the Meso-Neoproterozoic in the study area.
2. The Meso-Neoproterozoic sedimentary filling sequence structure in the Yanliao aulacogen is obvious. In the Mesoproterozoic, it experienced four stages : continental rift deposition (Changzhougou Formation-Tuanshanzi Formation), the transformation from continental rift to passive continental margin (Dahongyu Formation), passive continental margin deposition (Gaoyuzhuang Formation-Tieling Formation), active continental margin deposition (Xiamaling Formation ). The formation of the Qingbaikou system in the Neoproterozoic (starting from the Luotuoling Formation) is the result of a new continental extension, which is a passive continental margin stage. The Xiong 'er aulacogen has experienced three stages : the early continental rift stage (Xiong 'er Group ), the late continental rift stage (Xiaogoubei Formation-Yunmengshan Formation), and the passive continental margin stage (Baicaoping Formation-Huanglianduo Formation). The formation of the Qingbaikou System-Sinian System (starting from the Dongjia Formation) in the Neoproterozoic is a new passive continental margin stage.
3. The Yanliao and Xiong 'er aulacogens are both located in the North China Craton and formed in the Meso-Neoproterozoic, but their sedimentary filling sequences are obviously different, mainly in two aspects: (1) The formation time of each sedimentary filling sequence stage of different aulacogens is different; (2) The formation time of each sedimentary filling sequence stage of different aulacogens is different. There are obvious differences in rock characteristics, lithology combination, lithology structure, contact relationship, vertical sequence and sedimentary facies combination in the same sedimentary filling sequence stage. The filling characteristics of the two aulacogens completely record the geological events related to the breakup of the Colombian supercontinent.

## Data Availability

All data generated or analysed during this study are included in this published article [and its supplementary information files].
